# Intratumoral lactate metabolism in Barrett's esophagus and adenocarcinoma

**DOI:** 10.18632/oncotarget.15284

**Published:** 2017-02-11

**Authors:** Heikki Huhta, Olli Helminen, Sami Palomäki, Joonas H. Kauppila, Juha Saarnio, Petri P. Lehenkari, Tuomo J. Karttunen

**Affiliations:** ^1^ Departments of Pathology, University of Oulu, Oulu, Finland; ^2^ Departments of Surgery, University of Oulu, Oulu, Finland; ^3^ Medical Research Center Oulu, Oulu, Finland; ^4^ Oulu University Hospital, Oulu, Finland; ^5^ Upper Gastrointestinal Surgery, Department of Molecular Medicine and Surgery, Karolinska Institutet, Karolinska University Hospital, Stockholm, Sweden

**Keywords:** MCT1, MCT4, MTCO1, esophageal adenocarcinoma, Barrett's esophagus

## Abstract

**Background:**

Monocarboxylate transporters (MCTs) are cell membrane proteins which transport pyruvate, lactate and ketone bodies across the plasma membrane. MCTs are activated in various cancers, but their expression in esophageal adenocarcinoma is not known. The present study was conducted to elucidate the expression of MCTs in esophageal adenocarcinoma and its precursor lesions.

**Results:**

Cytoplasmic MCT1, MCT4 and MTCO1 expression linearly increased from normal epithelium to Barrett's mucosa to dysplasia and cancer. Low cytoplasmic MCT1 expression associated with high T-class (*P* < 0.01), positive lymph node metastases (*P* < 0.05), positive distant metastases (*P* < 0.01) and high tumor stage (*P* < 0.01). High cytoplasmic MCT4 expression correlated significantly with positive distant metastases (*P* < 0.05). Both low MCT1 and high MCT4 histoscore predicted survival in univariate analysis (*P* < 0.01). MCT4 histoscore predicted survival in multivariate analysis (*P* = 0.043; HR 1.8 95%CI 1.0–3.1). MTCO1 expression was not correlated to clinicopathological variables or survival.

**Materials and Methods:**

MCT1, MCT4 and mitochondrial cytochrome c oxidase (MTCO1) expression were determined with immunohistochemistry in esophageal specimens from 129 patients with columnar dysplasia or adenocarcinoma. Specimens including normal esophagus (*n* = 88), gastric (*n* = 67) or intestinal metaplasia (*n* = 51), low-grade (*n* = 42), high-grade dysplasia (*n* = 37) and esophageal adenocarcinoma (*n* = 99) were evaluated.

**Conclusions:**

Major increase in markers of tumor metabolism occurs during carcinogenesis and progression of esophageal adenocarcinoma. MCT1 and MCT4 are prognostic factors in esophageal adenocarcinoma.

## INTRODUCTION

The incidence of Esophageal adenocarcinoma is rising in the Western World, with low survival rates even after initially curative surgery [1]. Esophageal adenocarcinoma arises from Barrett's esophagus, which is considered a complication of long-term esophagitis due to reflux disease. Barrett's esophagus influence 2 to 7 per cent of adults in Western countries [2].

Normal cells rely on aerobic mitochondrial metabolism while cancer cells tend to produce energy through anaerobic glycolysis. Persistent activation of the glycolysis can favor aggressive proliferation, invasion and metastatic behavior [3, 4]. Mitochondrial energy metabolism profile, especially oxidative phosphorylation and glycolysis, undergoes various changes during the metaplasia–dysplasia–adenocarcinoma sequence in Barrett's esophagus. [5]. Monocarboxylate transporters (MCT) are cell membrane proteins allowing lactate to pass through cell membrane [6]. MCT family includes 14 members. MCT1-4 have been demonstrated to mediate proton-linked bi-directional transport of monocarboxylates such as lactate, pyruvate, and ketone bodies across the plasma membrane. Increased expression of MCT1 and MCT4 are reported in various cancers [7].

Alterations in markers of energy metabolism are poorly known in esophageal adenocarcinoma. The aim of this study was to assess the metabolic changes during development of esophageal adenocarcinoma by evaluating MCT1, MCT4 and mitochondrial cytochrome c oxidase (MTCO1) in different stages of esophageal metaplasia-dysplasia-adenocarcinoma-sequence.

## RESULTS

**Figure 1:**
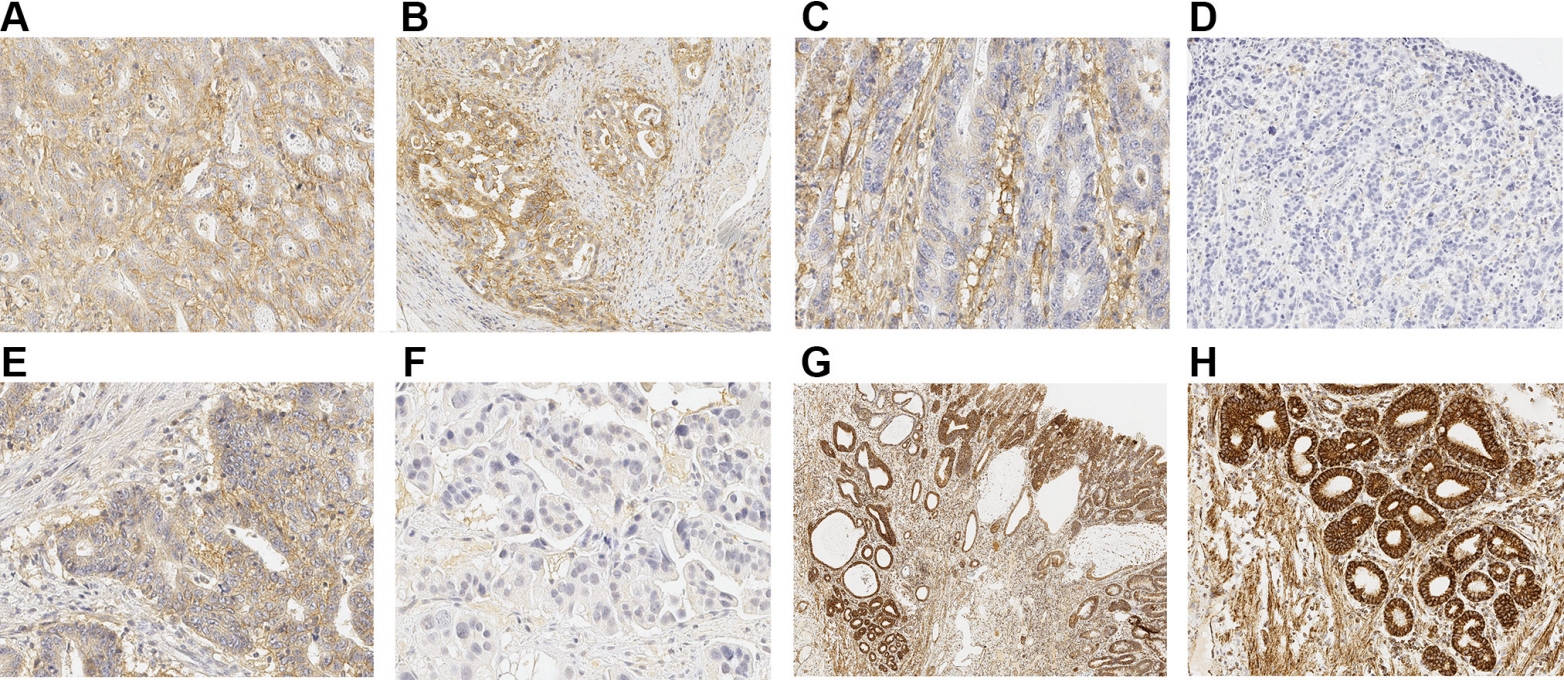
Immunohistochemical staining patterns of MCTs and MTCO1 (**A**) Strong immunoreaction of MCT4 in both cancer cells and in cancer stroma. Strong diffuse expression of MCT4 can be seen. (**B**) Cancer showing negative staining of MCT4 in tumor stroma but strong MCT4 expression in tumor cells. (**C**) MCT4 shows strong reaction in tumor stroma and only weak diffuse expression in cancer cells. (**D**) Adenocarcinoma negative for MCT4, neither tumor stroma nor cells express MCT4. (**E**) Strong MCT1 expression in cancer cells without stromal staining (**F**) Cancer negative for MCT1. (**G** and **H**) Parallel figure with 2× and 10× magnifications showing strong immunoreaction for MTCO1 in glandular structures of Barrett's dysplastic lesion and adenocarcinoma. Adenocarcinoma can be seen on the left, low-grade dysplasia in the middle and high-grade dysplasia on the right. (H) shows 10× magnification from adenocarcinoma in (G).

All available lesions were analyzed from the patient samples, but no more than one lesion of each type from a single patient. We thus analyzed 88 normal epithelial, 67 gastric- and 51 intestinal metaplasias, 42 low-grade- and 37 high-grade dysplasias and 99 adenocarcinomas. Majority of these lesions were from cancer patients. A total of 23 normal epithelia, 25 gastric- and 22 intestinal metaplasia and 30 low-grade- and 10 high-grade dysplasias were analyzed from patients with dysplasia as the most advanced diagnosis. MCT1, MCT4 and MTCO1 were all expressed in all studied tissues. Expression was predominantly cytoplasmic, and occasionally detected in cell membranes (Figure 1). Distribution of immunostaining was diffuse throughout the epithelium, except in normal squamous epithelium, where basal staining was observed.

### MCT 1, MCT4 and MTCO1 expression in non-dysplastic lesions

Normal epithelium showed weakest cytoplasmic expression in all studied markers. Cytoplasmic expression of MCT4 and MTCO1 was significantly higher in metaplastic cells compared to normal epithelium. Cytoplasmic expression of MCT1 was higher in intestinal metaplasia compared to normal squamous epithelium. MCT1 and MCT4 expression in the subepithelial stroma was significantly higher in metaplasia than in normal epithelium. MTCO1 expression was similar in both normal and metaplastic epithelial stroma. Histoscores for MCT1, MCT4 and MTCO1 in different lesions are summarized in Table 1.

**Table 1:** Baseline characteristics of MCT1, MCT4 and MTCO1 expression in normal esophageal squamous epithelium and in different esophageal lesions

	histoscore	histoscore	statistical	stroma	stroma	statistical
MCT1	mean	95% CI	significance	mean	95% CI	significance
Normal epithelium	45	35–55		0.7	0.5–0.9	
Gastric metaplasia	64	50–79		1.9	1.5–2.2	a
Intestinal metaplasia	101	78–125	a	1.5	1.2–1.8	a
Low-grade dysplasia	116	89–143	ab	1.8	1.4–2.2	a
High-grade dysplasia	149	117–181	ab	1.9	1.4–2.4	a
Adenocarcinoma	161	143–179	abc	1.4	1.1–1.6	a
MCT4						
Normal epithelium	27	17–24		0.5	0.3–0.6	
Gastric metaplasia	86	70–102	a	1.1	0.9–1.4	a
Intestinal metaplasia	86	63–109	a	1.6	1.3–1.9	a
Low-grade dysplasia	137	108	abc	2.0	1.7–2.4	ab
High-grade dysplasia	127	95–169	a	2.0	1.6–2.5	ab
Adenocarcinoma	148	127–170	abc	2.5	2.2–2.8	abc
MTCO1						
Normal epithelium	41	33–48		0.7	0.5–0.8	
Gastric metaplasia	107	94–120	a	1.0	0.8–1.2	
Intestinal metaplasia	147	128–165	ab	1.2	09–1.48	
Low-grade dysplasia	181	157–203	ab	1.3	1.0–1.6	a
High-grade dysplasia	209	182–235	abc	1.5	1.0–2.0	a
Adenocarcinoma	216		abc	1.4	1.2–1.7	a

a compared to normal epithelium, *p* < 0.05.

b compared to gastric metaplasia, *p* < 0.05.

c compared to intestinal metaplasia, *p* < 0.05.

Histoscore is counted by multiplying staining intensity (0–3) with the percentage of positive cells (0–100%), resulting with value between 0 and 300. Stromal staining was evaluated with 5 point scale (0–4). Values are presented as mean and 95% confidential interval (95%CI). For statistical testing One way ANOVA with Tukey in post hoc analysis was used.

### MCT 1, MCT4 and MTCO1 expression in dysplastic lesions and adenocarcinoma

Low- and high-grade dysplasia showed higher cytoplasmic expression of MCT1, MCT4 and MTCO1 than non-dysplastic lesions. MCT1 and MTCO1 expression increased towards high-grade dysplasia (Table 1). Rising stromal expression was observed in MCT4, whereas stromal expression of MCT1 and MTCO1 remained relatively stable.

Adenocarcinoma showed slightly higher cytoplasmic expression of MCT1, MCT4 and MTCO1 compared to dysplastic lesions. However, this difference was not statistically significant. Stromal MCT4 expression was the highest in adenocarcinoma, whereas expression of MCT1 and MCTO1 in tumor stroma did not significantly differ from other lesions. Cytoplasmic and stromal stainings are summarized in Table 1.

To explore the possible field-effects in the studied markers the expression of the studied markers in premalignant lesions was compared between cancer patients and dysplasia patients. The mean MCT1 histoscore was significantly higher in premalignant lesions of patients with adjacent carcinoma compared to patients with dysplasia as the most advanced lesion (gastric metaplasia 81 vs. 39, intestinal metaplasia 131 vs. 67 and low-grade dysplasia 148 vs. 81, all *p* < 0.05). No differences were observed in MCT4 and MTCO1 expression between carcinoma and dysplasia patients.

### MCT1, MCT4 and MTCO1 expression correlations with clinicopathological variables and cancer survival

**Figure 2:**
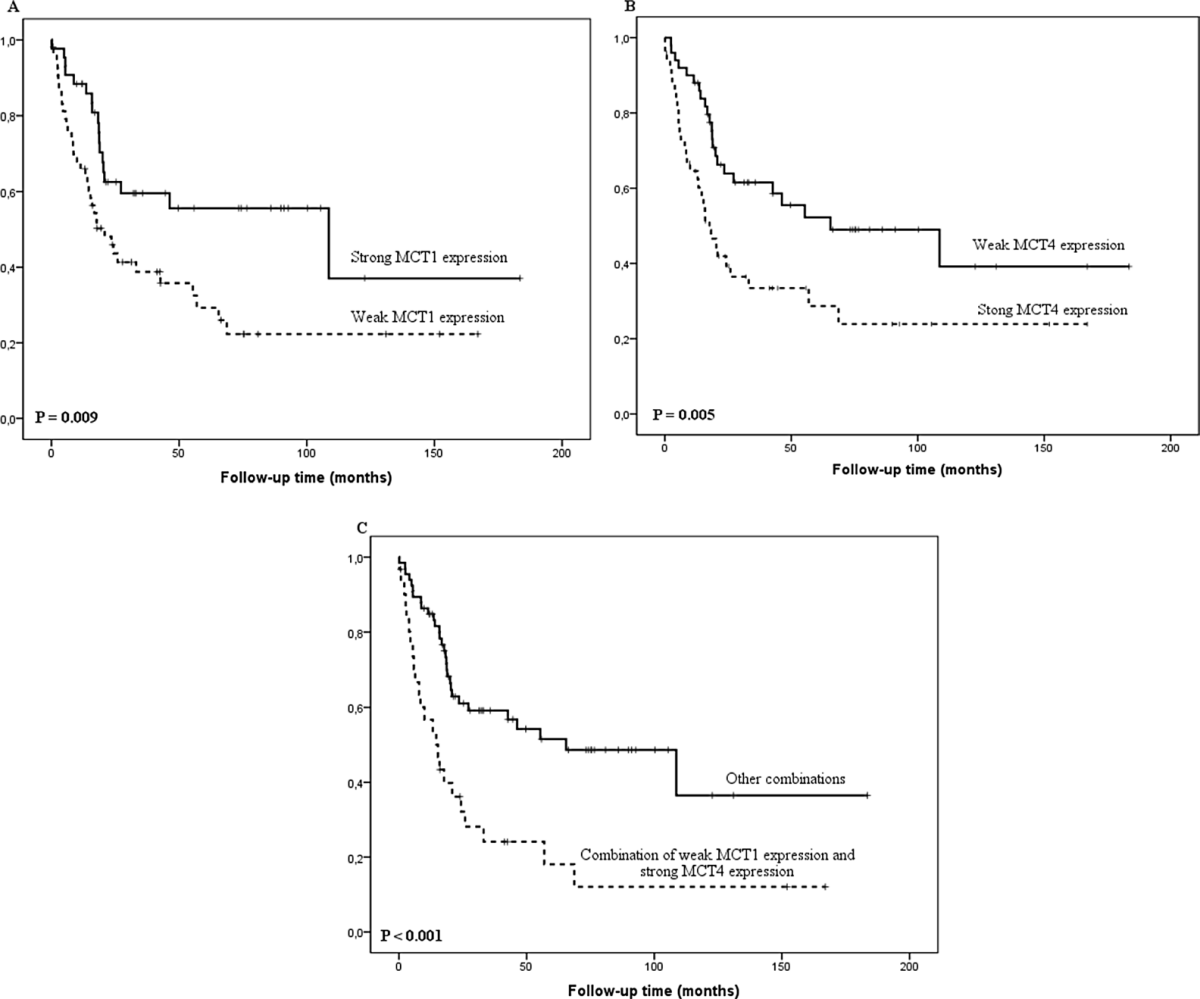
Kaplan-Meier curve showing esophageal adenocarcinoma survival stratified by MCT1 histoscore (**A**), MCT4 histoscore (**B**) and combination of low MCT1 and high MCT4 histoscore (**C**).

Low cytoplasmic MCT1 expression correlated statistically significantly with higher T-class (*P* = 0.002), positive lymph node metastases (*P* = 0.039), positive distant metastases (*P* = 0.006) and higher tumor stage (*P* = 0.009, Table 2). Low MCT1 histoscore predicted survival in univariate (*P* = 0.009, Figure 2), but not in multivariate analysis (data not shown).

**Table 2:** MCT1 and MCT4 histoscores compared to clinicopathological variables in esophageal adenocarcinoma

Variable	n/N	MCT1 histoscore, *n*			MCT4 histoscore, *n*			Combination of low MCT1 and high MCT4 histoscore		
		Low	High	*p*	Low	High	*p*	Others	Low MCT1, high MCT4	*p*
T										
T_1_	15/98	5	10	**0.002**	9	6	0.798	12	3	0.195
T_2_	14/98	4	8		8	6		10	2	
T_3_	53/98	29	24		25	28		36	17	
T_4_	16/98	15	1		8	8		8	8	
Lymph nodes										
negative	37/98	15	21	**0.039**	23	14	0.086	29	7	0.053
positive	61/98	38	22		27	34		37	23	
Organ metastases										
negative	66/98	29	35	**0.006**	39	27	**0.022**	52	12	**< 0.001**
positive	32/98	24	8		11	21		14	18	
Grade										
1	29/98	11	16	0.112	17	12	0.353	21	6	0.508
2	26/98	13	13		10	16		16	10	
3	43/98	29	14		22	21		28	15	
Stage										
I	15/98	3	12	**0.009**	10	5	0.059	14	1	**0.003**
II	37/98	18	17		20	17		27	8	
III	13/98	9	4		9	4		10	3	
IV	33/98	23	10		11	22		15	18	
Tumor size										
small (< 40 mm)	39/95	20	18	0.722	25	14	**0.042**	30	8	0.080
large (≥ 40 mm)	56/95	31	24		24	32		34	21	

Combination variable for low MCT1 and high MCT4 histoscore was counted. TNM-staging and grade of differentiation was available from 98 patients and tumor size from 95 patients. MCT1 material consisted only of 97 patients due to unrepresentative samples. Significant *p*-values are shown in bold.

High cytoplasmic MCT4 expression correlated significantly with positive distant metastases (*P* = 0.022) and large tumor size (*P* = 0.042, Table 2). MCT4 histoscore predicted survival in both univariate (*P* = 0.005, Figure 2), and in multivariate analysis (*P* = 0.043; HR 1.8 95%CI 1.0–3.1, Figure 2).

Since low cytoplasmic MCT1 and high MCT4 expression correlated with clinicopathological variables and survival, we analyzed cancers with low MCT1 expression (histoscore ≤ 150) and high MCT4 expression (histoscore > 110) combined. This combination value showed significant correlation with distant metastasis (*P* < 0.001) and tumor stage (*P* = 0.003, Table 2). The combination predicted survival in univariate analysis (*P* < 0.001, Figure 2). Multivariate analysis showed a borderline statistical significance for worse survival in MCT1-/MCT4+ cancers (*P* = 0.059; HR 1.8 95%CI 1.0–3.2).

Cytoplasmic MTCO1 expression was not correlated to any of the clinicopathological variables or survival (data not shown).

### Analysis of intensity and percentage of positive cells separately from histoscore

We also evaluated intensity and percentage of MCT1, MCT4 and MTCO1 separately without histoscore. Low cytoplasmic MCT1 expression correlated statistically significantly with higher T-class (*P* = 0.002), positive lymph node metastases (*P* = 0.039), positive distant metastases (*P* = 0.006) and higher tumor stage (*P* = 0.009, Table 2). Percentage of MCT1 positive cells did not correlate to studied clinicopathological variables or survival. High cytoplasmic MCT4 intensity and high percentage of positive cells correlated significantly with distant metastases (intensity *P* = 0.010; percentage *P* = 0.032) and with high tumor stage (intensity *P* = 0.033; percentage *P* = 0.035). MTCO1 percentage of positive cells correlated significantly with poor grade of differentiation.

Stromal expression of MCT1, MCT4 or MTCO1 showed no correlation with any clinical parameters of survival.

## DISCUSSION

In this study we characterized MCT1, MCT4 and MTCO1 expression in esophageal metaplasia–dysplasia–adenocarcinoma sequence. Epithelial cytoplasmic MCT- and MTCO1 expression linearly increased towards dysplasia and adenocarcinoma. Weak cytoplasmic MCT1 expression and strong MCT4 expression correlated to metastases and poor prognosis in esophageal adenocarcinoma.

The Warburg effect has been suggested to be a result of metabolic collaboration between cancer cells and stroma. Cancer cells form a mitochondrially active region while glycolytic stroma shuttles metabolites to cancer cell supporting growth and invasion [8, 9–11]. MCTs transport high-energy metabolites through cell membrane and regulate the pH of the tumor microenvironment [7]. Lactate-extruding MCT4 is induced by hypoxia, and is a known HIF1-alpha target gene [12, 13] In contrast, MCT1 transporter facilitates the uptake of lactate [6]. Our data shows significant increase of MCT expression (associated with higher MTCO1 expression) in Barrett's dysplasia compared to normal epithelium indicating increased metabolic activity in these lesions. Stepwise increase in metaplasia–dysplasia–adenocarcinoma sequence was observed with all studied immunostainings. The mean expression of MCT1 and MCT4 decreased from dysplastic lesions to adenocarcinoma. These findings suggest that a complex metabolic shift occurs during metaplastic and dysplastic changes in the esophagus.

MCT1 has been previously reported to be highly expressed in central nervous system, breast, lung, cervix, prostate and stomach cancers [14–19]. High MCT4 expression has been shown to correlate to poor prognosis in breast, lung, gastric, colon and prostate cancer [17, 20–22]. In this study, low cytoplasmic MCT1 expression and high MCT4 expression in cancer cells correlated to advanced stages and survival. Furthermore, the cytoplasmic MCT1-/MCT4+ combination score correlated significantly with distant metastases and tumor stage. Mitochondrial energy metabolism is altered in metaplasia-dysplasia-adenocarcinoma sequence. Oxidative phosphorylation profiles might predict progression of Barrett's esophagus to adenocarcinoma [5]. MTCO1 is a core component in cytochrome c oxidase, which is terminal enzyme of the respiratory electron transport chain of mitochondria [23]. We showed that expression increased during esophageal adenocarcinoma carcinogenesis with the highest MTCO1 expression in adenocarcinoma indicating increased activity of aerobic mitochondrial energy metabolism. Our data suggests that metabolic profile is altered in Barrett's esophagus and esophageal adenocarcinoma. However, MTCO1 expression did not correlate to clinical outcome. Similar changes in mitochondrial energy metabolism have been previously reported in for example prostate, neck/head and breast cancers but in these cancers, the changes were associated with poor prognosis [8–10, 24]. There are also opposite findings, for example in renal cell carcinoma [25].

Our findings do not fit in to the theory of “metabolic coupling”, but there are other models of lactate shuttle in cancer [7]. Our data suggests that lactate is shuttled out from cancer cells for controlling pH and as a signaling molecule to support for instance cell migration and tumor angiogenesis [7]. Our observation is also supported by a report of increased activity of carbonic anhydrase IX in esophageal adenocarcinoma and its correlation to poor survival. This indicates important role of pH control in esophageal adenocarcinoma [26]. Gram-negative bacteria tolerate acidic environments [27] and bacterial flora undergoes a shift towards more gram-negative flora in Barrett's esophagus [28, 29]. This might be related to lactate metabolism. However, with the strong causal relationship between acid reflux and Barrett's esophagus, the relationship is likely complex.

Strength of the current study is that the treatment of all esophageal cancer patients in Northern Finland is centralized solely to Oulu University Hospital, making our study less prone to selection bias. Interobserver agreement was excellent with no required consensus statements, indicating fluent repeatability of the evaluation. Use of only immunohistochemistry is a possible weakness. The immunohistochemical analysis was validated via dual negative controls and tested for the effect of the age of the paraffin blocks. We tested the markers for field-effect by comparing the expression levels in normal, metaplastic and dysplastic epithelium between adenocarcinoma and dysplasia patients [30]. There were no indication for such effect for MCT4 and MTCO1. MCT1 expression was higher in patients with esophageal adenocarcinoma compared to those with dysplasia as the most advanced diagnosis, suggesting a possible field-effect by cancer. However, the expression of MCT1 similarly increased during metaplasia-dysplasia sequence in both groups. The observed possible field-effect thus does not bias the interpretation of the study results.

In conclusion, the expression of MCT1, MCT4 and MTCO1 increase from Barrett's esophagus to dysplasia indicating metabolic alteration during dysplastic progression. Low cytoplasmic expression of MCT1 and high expression of MCT4 associate with advanced stage and poor prognosis in esophageal adenocarcinoma.

## MATERIALS AND METHODS

### Patients

The use of patient samples and the data inquiry were approved by the Oulu University Hospital Ethics Committee. The need to obtain a written or oral consent from the patients for using the samples in research was waived by the Finnish National Authority for Medicolegal Affairs (VALVIRA, Dnro 10832/06.01.03.01/2014)

Paraffin-embedded, archival specimens of esophageal adenocarcinoma or esophageal dysplasia were obtained from the Department of Pathology, Oulu University Hospital, between the years 1987–2013. The final series consisted of 99 patients with esophageal adenocarcinoma, 10 with high-grade dysplasia, and 20 with low-grade dysplasia as the most advanced lesion. The specimens with carcinoma or dysplasia contained a selection of other tissues and lesions, such as normal esophageal mucosa, gastric or intestinal metaplasia and less advanced dysplasia, these being present in either the same tissue block or additional tissue blocks. The material has been earlier described elsewhere [31, 32]. The median age of the cancer patients was 64 years (range 43–90). The median follow-up time was 36 months (range 0–288 months) for the surviving patients. The patient survival data was acquired from Statistics Finland. TNM-staging and grade of differentiation was available from 98 patients and tumor size from 95 patients. MCT1 immunohistochemical material consisted only of 97 adenocarcinoma samples due to unrepresentative samples.

### Immunohistochemistry

Immunohistochemistry was performed on the tissue block sections, which were first selected by expert gastrointestinal pathologist, on the basis of hematoxylin and eosin-staining, to be representative for the tumor mass in the resected specimen. Dako Envision kit (Dako, Copenhagen, Denmark) was used for immunohistochemical with a high temperature antigen retrieval in citrate buffer for 15 minutes. Diaminiobenzidine (Dako basic DAB-kit) was used as a chromogen. All staining was done with Dako Autostainer (Dako, Copenhagen, Denmark). Immunostaining was performed with a commercial antibody MCT1; sc-50324, rabbit polyclonal IgG, lot I2710, dilution 1:100, MCT4; sc-50329, rabbit polyclonal IgG, lot A3113 dilution 1:500, Santa Cruz Biotechnology, Dallas, Texas, US and MTCO1; ab14705, mouse monoclonal IgG2a, lot GR94203-16, dilution 1:100, Abcam, Cambridge, UK

We validated the immunohistochemical analysis two series of negative controls (omitting the primary antibody and by replacing primary antibody with the mouse primary antibody isotype control). To confirm the antigen preservation in the old paraffin blocks we compared the MCT1, 4 and MTCO1 staining intensities in esophageal adenocarcinoma between old and new blocks divided by the median age of the blocks. No significant differences were found.

### Assessment of immunostaining

The hematoxylin and eosin-staining sample slides were digitized using Aperio AT2 Console, Leica Biosystems Imaging Inc, Nussloch, Germany for identification and marking of different lesions in the specimens. Identification was made an expert gastrointestinal pathologist (T.J.K.). Immunoreactivity of MCT1, 4 and MTCO1 was analyzed by two independent researchers (H.H and O.H) who were blinded from the clinical data, using method described earlier. We assessed the intensity of staining (0–3), the percentage of positive cells (0–100), the percentage of nuclear and membrane positive cells (0–100) [31, 32]. Tumor stromal staining pattern assessed as 0, no detectable staining; 1, focal staining; 2, areas diffuse staining present in less than half of stromal area; 3, expression of moderate density distributed in more than half but not in all parts of the tumor stroma; 4, dense expression extending throughout the stroma as previously described [8]. All evaluated parameters were assessed in normal esophageal squamous epithelium, gastric and intestinal metaplasia, low- and high-grade dysplasia and adenocarcinoma. Mean values of two independent estimates were used if there was no difference over 1 in the intensity or over 30% in the percentage. If the difference was more extensive, consensus was reached after re-evaluation with a third researcher (T.J.K). Evaluation did not differ as cross-borders was set and for that re-evaluation not needed. Mean intensity and mean percentage was then multiplied together to obtain a histoscore (0–300). Histoscore was dichotomized into equally sized groups by the median value of MCT1, MCT4 and MTCO1 histoscore as previously described [33, 34].

### Statistical analysis

We used IBM SPSS Statistics 22.0 (IBM corp., Armonk, NY) for statistical analyses. To compare immunostainings expression between different lesions we used one way ANOVA with Tukey in post hoc analysis was used. Independent sample *T*-test was used to compare histoscores between carcinoma patients (*n* = 99) and patients with dysplasia (*n* = 30, HGD and LGD combined) to evaluate possible field-effect of adjacent adenocarcinoma. The chi-square-test was used to calculate statistically significant differences between prognostic and clinicopathologic variables. Life tables were calculated according to the Kaplan-Meier method, and the survival curves were compared using the log-rank test. Cox proportional hazards model with backward selection was used for multivariate analysis with following covariates: Age, gender, T-stage, N-stage, M-stage and grade of differentiation.
